# Supplementary material for: Sequence Length of HIV-1 Subtype B Increases over Time: Analysis of a Cohort of Patients with Hemophilia over 30 Years
Source: Viruses. 2021 Apr 30;13(5):806. doi: 10.3390/v13050806 (PMC8145643; doi:10.3390/v13050806)
Supplement: Supplementary file 1 [file viruses-13-00806-s001.zip › Table S1.pdf]

Table S1. Characteristics of the 62 patients determined near full-length sequences of KSB HIV-1 and 3 non-KSB patients.

Patient (code) or isolate	Year of diagnosis	Date of sample collection (date-year)	Near full length sequences	GenBank no.	CD4+ T cell/ul	Viral load (copy/ml)	*Env* gene sequence length	*nef* gene sequence length	Duration of cART (mo)
141 Korean subtype B									
93BGO2	1988	Feb-93	8,651	MT559045	256	2,393	2,595	621	0
92HSH1	1989	Jan-92	8,645	MK577481	594	873	2,589	621	0
92LSW5	1989	May-92	8,638	KJ140266	329	34,355	2,592	621	0
92LSY12	1989	Dec-92	8,606	MN043589	279	583	2,550	621	0
00LJI12	1989	Dec-00	8,624	MN043590	53	90,467	2,568	621	12
93PJA9	1989	Sep-93	8,696	MN043595	124	20,808	2,613	648	0
92KJS11	1989	Nov-92	8,696	MN043596	242	1,703	2,613	648	0
04KJS8	1989	Aug-04	8,687	JQ316130	109	162,000	2,568	651[b]	0
92KYJ5	1989	May-92	8,654	MN043597	315	12,748	2,598	621	0
93KHS5	1989	May-93	8,666	MN043593	138	64,405	2,589	642	0
93HYH5	1989	May-93	8,603	MN043594	266	995	2,547	621	0
99HYH2	1989	Feb-99	8,636	JQ316129	18	211,506	2,580	621	0
93BJR3	1989	Mar-93	8,648	MN043586	558	995	2,592	621	0
92LSK6	1990	Jun-92	8,638	MN043582	279	1,181	2,582	621	0
04LSK7	1990	Jul-04	8,667	DQ295192	225	4,500	2,589	624	0
92KChS3	1990	Mar-92	8,505 [c]	MT559044	387	ND	2,536[c]	624	0
92YWS6	1990	Jun-92	8,600	MK577480	307	1,292	2,544	621	0
93LSH5	1990	May-93	8,600	MN043583	813	1,925	2,547	621	0
03LSH1	1990	Jan-03	8,693	JQ316127	501	116,000	2,619	630	0
92KHB4	1990	Apr-92	8,612	MN043584	252	1,732	2,556	624	0
94KHB5	1990	May-94	8,639	KJ140267	208	6,307	2,583	621	0
93KDG4	1990	Apr-93	8,597	MN237642	319	408	2,541	621	0
93JJS10	1990	Oct-93	8,654	MK577479	436	360	2,598	621	0
93YEH1	1990	Jan-93	8,618	MN043592	418	3,689	2,562	621	0
03YGS3	1990	Mar-03	8,675	JQ316135	578	698	2,619	621	0
93KJin2	1990	Feb-93	8,594	MN043581	833	37,728	2,565	621	0
04KJin8	1990	Aug-04	8,654	DQ295195	332	104,000	2,604	621	0
92OCH3	1990	Mar-92	8,657	MN237644	247	2,790	2,589	633	0
91LCS9	1991	Sep-91	8,614	MT559066	1620	6,310	2,553	627	0
92DGi6	1991	Jun-92	8,618	MT559046	552	12,385	2,562	621	0
92JWK3	1991	Mar-92	8,633	MN043585	422	7,870	2,577	621	0
97JWk7	1991	Jul-97	8,657	AF224507	701	5,825	2,601	621	0
04JWk7	1991	Jul-04	8,612	DQ295194	642	17,800	2,553	621	0
91HJY7	1991	Jul-91	8,639	MN043577	337	1,470	2,574	621	0
03HJY8	1991	Aug-03	8,651	JQ316131	238	1,700	2,595	621	0
91KGS7	1991	Jul-91	8,613	MN043576	453	7,870	2,554	621	0
99KGS8	1991	Aug-99	8,693	MT877449	95	ND	2,607	648	0
03KGS5	1991	May-03	8,681	JQ316132	15	366,197	2,496	648	15
91KYB12	1991	Dec-91	8,615	MN043591	137	434,445	2,559	621	0
92HJiH1	1991	Jan-92	8,605	MN043588	533	16,044	2,558	621	0
Donor O:91OSG10	1990	Oct-91	8,609	KF561442	409	7,180	2,553	621	0
Donor O:02OSG1	1990	Jan-02	8,627	JQ429433	216	97,800	2,571	621	36
HP-1:92Lys9	1990	Sep-92	8,618	KJ140245	425	2,515	2,562	621	0
HP-2:92JJW1	1991	Jan-92	8,624	KJ140247	636	34,336	2,568	621	0
HP-2:04JJW2	1991	Feb-04	8,663	MT679550	261	35,900	2,607	621	0
	1991	Feb-04	8,666	MT679551	261	35,900	2,610	621	0
HP-2:19JJW6	1991	Jun-19	8,616	MT559047	380	<20	2,604	621	156
HP-2:20JJW4	1992	Apr-20	8,604 [a]	MW881616	444	<20	2,592	621	166
HP-3:92LJW8	1991	Aug-92	8,627	KJ140248	365	22,370	2,571	621	0

HP-3:02LJW8	1991	Aug-02	8,645	MT582420	26	107,800	2,589	621	0
HP-3:12LJW12	1991	Dec-12	8,662	MW405324	471	128	2,610	618	117
HP-3:16LJW2	1991	Feb-16	8,677	MT224125	679	<20	2,616	621	156
HP-3:20LJW2	1991	Feb-20	8,716	MT559048	757	<20	2,643	618	204
HP-3:20LJW2	1991	Feb-20	8,704	MT559049	757	<20	2,643	621	204
HP-3:21LJW2	1991	Feb-21	8,680 [a]	MW881620	840	<20	2,624	621	216
HP-3:21LJW2	1991	Feb-21	8,674 [a]	MW881619	840	<20	2,618	621	216
HP-3:21LJW2	1991	Feb-21		MW881621	840	<20		621	216
HP-4:93KGJ10	1991	Oct-93	8,621	KU869610	826	<8,180	2,565	621	0
HP-4:03KGJ2	1991	Feb-03	8,709	KJ140249	319	8,180	2,629	645	0
HP-4:09KGJ12	1991	Dec-09	8,636[a]	MW405267	539	<20	2,580	621,651 [b]	73
HP-4:19KGJ7	1991	Jul-19	8,657	MT559050	914	<20	2,601	621	192
HP-6:92JHJ9	1992	Sep-92	8,606	KJ140251	881	3,849	2,550	621	0
HP-6:03JHJ2	1992	Feb-03	8,693	MT679552	690	<20	2,640	618	0
HP-6:19JHJ7	1992	Jul-19	8,672	MT559051	195	<20	2,619	618	222
HP-8:92CWS5	1992	May-92	8,596 [c]	MN043599	241	1,120	2,553	621	0
HP-8:95CWS4	1992	Apr-95	8,621	KJ140253	190	<3,765	2,565	621	0
HP-8:03CWS10	1992	Oct-03	8,652	MN043600	184	14,600	2,586	630	0
HP-8:04CWS5	1992	May-04	8,543 [c]	JQ316133	212	ND	2,478[c]	630	0
HP-8:09CWS12	1992	Dec-09	8,709	KU869620	120	<20	2,646	ND	28
HP-8:11CWS1	1992	Jan-11	8,654	MT582421	35	2,080	2,601	630	48
HP-8:11CWS1	1992	Jan-11	8,654[a]	MW405272	35	2,080	2,601	630	48
HP-8:11CWS1	1992	Jan-11	8,654[a]	MW405271	35	2,080	2,601	630	48
HP-10:92SHJ8	1992	Aug-92	8,597	KJ140255	142	11,509	2,541	621	0
HP-10:02SHJ8	1992	Aug-02	8,663	MN043601	36	12,020	2,589	639	0
HP-18:93JHS5	1993	May-93	8,612	KF561443	191	23,818	2,556	621	0
HP-18:97JHS8	1993	Aug-97	8,630	MW405327	21	20,960	2,574	621	0
HP-18:97JHS8	1993	Aug-97	8,639	MW405328	21	20,960	2,574	621	0
HP-18:07JHS10	1993	Oct-07	8,633	KJ140263	217	<20	2,577	621	118
HP-18:09JHS5	1993	May-09	8,636	MT559061	121	<20	2,571	621	137
Donor P	1991	Oct-93	8,618	MK577478	433	10,404	2,559	624	0
HP-5:92PJH8	1992	Aug-92	8,612	KJ140250	554	47,255	2,553	624	0
HP-5:95PJH6	1992	Jun-95	8,606	MT224126	98	15,701	2,550	621	0
HP-7:92LSM10	1992	Oct-92	8,606	KJ140252	780	5,297	2,550	624	0
HP-7:96LSM10	1992	Oct-96	8,669	MT679553	72	>86,508	2,598	636	0
HP-7:19LSM8	1992	Aug-19	8,654	MT559052	488	<20	2,580	639	132
HP-7:19LSM8	1992	Aug-19	8,672	MT559053	488	<20	2,601	636	132
HP-9:02KDE12	1992	Dec-02	8,663	KJ140254	281	11,500	2,604	624	0
HP-9:03KDE11	1992	Nov-03	8,660	JQ316128	502	11,500	2,604	621	0
HP-9:06KDE9	1992	Sep-06	8,657	MW405273	141	23,700	2,601	621	0
HP-9:07KDE12	1992	Dec-07	8,697 [a]	MW405274	492	<400	2,641	621	5
HP-9:07KDE12	1992	Dec-07	8,658 [a]	MW405275	492	<400	2,602	621	5
HP-9:07KDE12	1992	Dec-07	8,648	MW405325	492	<400	2,592	621	5
HP-9:11KDE3	1992	Mar-11	8,681	KU869551	650	<40	2,625	621	44
HP-9:11KDE3	1992	Mar-11	8,672	KU869552	650	<40	2,616	609	44
HP-9:18KDE11	1992	Nov-18	8,627	MT559054	1159	<20	2,583	609	132
HP-9:18KDE11	1992	Nov-18	8,675	MT559055	1159	<20	2,619	621	132
HP-11:92PGU7	1992	Jul-92	8,636	KJ140256	966	7,546	2,568	624	0
HP-11:02PGU10	1992	Oct-02	8,654	MN043602	414	80,500	2,574	636	0
HP-11:08PGU7	1992	Jun-08	8,710	KU896082	407	17,600	2,625	636	55
HP-11:08PGU7	1992	Jun-08	8,681	KU896083	407	17,600	2,601	636	55
HP-11:19PGU11	1992	Nov-19	8,663	MT559056	1277	<20	2,583	636	192
HP-11:19PGU11	1992	Nov-19	8,663	MT559057	1277	<20	2,583	636	192
HP-11:21PGU2	1992	Feb-21	8,666 [a]	MW881633	1909	<20	2,586	636	207
HP-11:21PGU2	1992	Feb-21	8,654 [a]	MW881634	1909	<20	2,574	636	207
HP-12:92LGH5	1992	May-92	8,606	KJ140257	767	26,616	2,547	624	0
HP-12:02LGH10	1992	Oct-02	8,648	MN043603	393	<400	2,592	621	5

HP-12:11LGH8	1992	Aug-11	8,654	KU869555	884	<20	2,598	609	111
HP-12:17LGH9	1992	Sep-17	8,642 [a]	MW405279	380	<20	2,586	ND	184
HP-12:19LGH11	1992	Nov-19	8,651	MT559058	384	<20	2,595	624	210
HP-12:19LGH11	1992	Nov-19	8,645	MT559059	384	<20	2,598	609	210
HP-12:21LGH3	1992	Mar-21	8,660 [a]	MW881635	367	<20	2,601	621	226
HP-13:92KMK7	1992	Jul-92	8,609	KJ140258	446	558	2,550	624	0
HP-13:04KMK5	1992	May-04	8,648	JQ316126	424	4,000	2,580	624	0
HP-13:14KMK2	1992	Feb-14	8,671	MW405326	377	<20	2,589	639	96
HP-14:92KTG3	1992	Mar-92	8,609	MN043604	420	<20	2,550	624	0
HP-14:97KTG8	1992	Aug-97	8,609	KJ140259	219	4,606	2,550	624	0
HP-14:07KTG10	1992	Oct-07	8,669	MN043605	47	88,600	2,592	630	0
HP-14:12KTG8	1992	Aug-12	8,686	KU869559	56	2,900	2,615	630	40
HP-14:19KTG8	1992	Aug-19	8,675	MT582422	202	<20	2,604	624	128
HP-14:21KTG3	1992	Mar-21	8,654 [a]	MW881636	115	<20	2,583	624	147
HP-15:03Lsw3	1992	Mar-03	8,642	KJ140260	643	111	2,556	651 [b]	0
HP-15:17Lsw1	1992	Jan-17	8,658 [a]	MW881637	85	nT	2,572	ND	60
HP-15:19Lsw12	1992	Dec-19	8,612	MT559060	75	<20	2,556	621	92
HP-16:92JIS12	1992	Dec-92	8,639	KJ140261	315	335	2,580	624	0
HP-16:03JIS10	1992	Oct-03	8,675	MN043606	179	14,280	2,610	630	0
HP-16:03JIS10	1992	Oct-03	8,672	KU869563	179	14,280	2,607	630	0
HP-16:12JIS11	1992	Nov-12	8,642	MT582423	425	<20	2,580	627	82
HP-16:16JIS2	1992	Feb-16	8,642 [a]	MW405280	501	<20	2,580	624	141
HP-17:93LSP3	1993	Mar-93	8,657	KJ140262	612	>3,703	2,601	621	0
HP-17:02LSP11	1993	Nov02	8,660	MN043607	66	<3,703	2,604	621	17 on Td
HP-17:18LSP12	1993	Dec-18	8,677	MT582424	313	4,324	2,616	621	209
HP-17:18LSP12	1993	Dec-18	8,640 [a]	MW881638	313	4,324	2,579	621	209
HP-17:18LSP12	1993	Dec-18	8,631 [a]	MW881639	313	4,324	2,570	621	209
HP-17:21LSP3	1993	Mar-21	8,663 [a]	MW881640	377	<20	2606	621	236
HP-17:21LSP3	1993	Mar-21	8,666 [a]	MW881641	377	<20	2609	621	236
HP-19:02LGS11	1993	Nov-02	8,639	KJ140264	352	1,721	2,550	636	18
HP-19:19LGS8	1993	Aug-19	8,615	MT559062	1076	<20	2,544	636	219
HP-19:19LGS8	1993	Aug-19	8,615	MT559063	1076	<20	2,544	636	219
HP-19:21LGS1	1993	Jan-21	8,619 [a]	MW881642	888	M20	2,547	636	236
HP-20:02KJO10	1994	Oct-02	8,711	KJ140265	219	12,000	2,607	621	78
HP-20:19KJO12	1994	Dec-19	8,702	MT559064	344	<20	2,616	624	206
HP-20:19KJO12	1994	Dec-19	8,699	MT559065	344	<20	2,613	621	206
HP-20:21KJO2	1994	Feb-21	8,693 [a]	MW881645	307	<20	2,607	621	220
92KSS12	1992	Dec-92	8,633	MN043587	490	12,119	2,574	624	0
92CYK6	1992	Jun-92	8,669	MN043598	176	11,642	2,613	621	0
92JSH3	1992	Mar-92	8,657	MT101871	387	48,018	2,583	629	0
92KYY3	1992	Mar-92	8,618	MN043579	975	21,341	2,553	630	0
96KYY1	1992	Jan-96	8,618	MT877450	1155	ND	2,553	630	0
96KYY1	1992	Jan-96	8,633	MT877451	1155	ND	2,568	630	0
07KYY4	1992	Apr-07	8,633	JQ341411	921	2,001	2,568	630	0
12KYY10	1992	Oct-12	8,633	KF561440	805	9,600	2,568	630	0
12KYY10	1992	Oct-12	8,654	KF561441	805	9,600	2,589	630	0
13KYY2	1992	Feb-13	8,660	MW405286	919	7,780	2,595	630	0
13KYY2	1992	Feb-13	8,672	MW405285	919	7,780	2607	630	0
13KYY7	1992	Jul-13	8,645	MK871374	864	10,000	2,580	630	0
14KYY6	1992	Jun-14	8,696	MT877455	855	14,900	2,631	630	0
17KYY5	1992	May-17	8,651	MG461319	778	34,000	2,586	630	0
	1992	May-17	8,693	MG461320	778	34,000	2,628	630	0
17KYY10	1992	Oct-17	8,660	MG461321	568	18,845	2,595	630	0
	1992	Oct-17	8,663	MG461322	568	18,845	2,598	630	0
19KYY9	1992	Sep-19	8,685	MT877452	836	<20	2,619	630	19
20KYY11	1992	Nov-20	8,667 [a]	MW405288	1113	<20	2601	630	33
93JIJ6	1992	Jun-93	8,660	MN237646	322	5,716	2,598	630	0
Donor R	1992	Jun-92	8,657	MN237644	511	14,748	2,589	633	0
93KJK4	1992	Apr-93	8,668	MN237645	177	5,230	2,613	621	0

93KYR2	1993	Feb-93	8,603	MN043580	476	11,318	2,544	624	0
04KYR8	1993	Aug 04	8,633	DQ295196	343	31900	2,571	624	0
04KMH5	1993	May-04	8,639	DQ295193	454	14,655	2,562	642	0
05YJN2	1996	Feb-05	8,648	JQ316134	640	29,200	2,586	627	0
04LHS6	2001	Jun-04	8,630	AY839827	566	1,300	2,574	627	48
28 Non-KSB subtypes									
91CSR4	1987	Apr-91	8,609	MW881608	384	ND	2,553	612-621	0
99CSR10	1987	Oct-99	8,669	MW405290	105	22,452	2,613	612	0
	1987	Oct-99	8,669 [a]	MW405291	105	22,452	2,613	612	0
00CSR3	1987	Mar-00	8,675 [a]	MW881646	136	10,838	2,619	612	0
	1987	Mar-00	8,627 [a]	MW881647	136	10,838	2,571	612	0
	1987	Mar-00	8,690 [a]	MW881648	136	10,838	2,634	612	0
04CSR10	1987	Oct-04	8,678 [a]	MW881649	189	26,900	2,622	612	0
05CSR3	1987	Mar-05	8,657	DQ837381	117	26,900	2,606	612	0
07CSR1	1987	Jan-07	8,699	MW405330	126	20,475	2,652	612	0
	1987	Jan-07	8,699 [a]	MW405293	126	20,475	2,652	612	0
	1987	Jan-07	8,675 [a]	MW405294	126	20,475	2,628	612	0
11CSR3	1987	Mar-11	8,738	MW405332	119	73,800	2,685	618	0
11CSR3	1987	Mar-11	8,714	MW405331	119	73,800	2,661	618	0
12CSR12	1987	Dec-12	8,633	MW405333	225	179,800	2,580	618	0
15CSR6	1987	Jun-15	8,594	MW405335	3	69,600	2,557	618	0
15CSR6	1987	Jun-15	8,657	MW405334	3	69,600	2,618	618	0
15CSR6	1987	Jun-15	8,621	MW405296	3	69,600	2,583	618	0
15CSR8	1987	Aug-15	8,603	MW405336	2	ND	2,580	615	0
15CSR8	1987	Aug-15	8,645 [a]	MW405298	2	ND	2,622	615	0
16CSR7	1987	Jul-16	8,630	MW405337	135	<20	2,580	615	11
93MHI10	1988	Oct-93	8,598	MW405338	913	<400	2,547	621	0
04MHI10	1988	Oct-04	8,609	JQ316137	574	821	2,547	621	0
07MHI10	1988	Oct-07	8,612	JQ316136	574	49,800	2,550	621	0
08MHI11	1988	Nov-08	8,606	JQ316138	1,154	ND	2,544	621	0
12MHI6	1988	Jun-12	8,636	MT877447	504	5,240	2,574	621	0
12MHI11	1988	Nov-12	8,636	KF561436	640	ND	2,574	621	0
12MHI11	1988	Nov-12	8,663	KF561437	640	ND	2,601	621	0
13MHI6	1988	Jun-13	8,633	KF561438	382	12,200	2,571	621	0
13MHI6	1988	Jun-13	8,633	KF561439	382	12,200	2,571	621	0
19MHI12	1988	Dec-19	8,636	MT877448	1,149	<20	2,602	621	76
94KBH10	1992	Oct-94	8,615	MW881610	280	781	2,574	609	0
95KBH4	1992	Apr-95	8,606	MW405339	344	781	2,565	609	0
95KBH4	1992	Apr-95	8,615	MW405340	344	781	2,574	609	0
01KBH3	1992	Mar-01	8,576	MW405341	450	1,571	2,535	609	0
01KBH7	1992	Jul-01	8,569	MW881609	892	1,571	2,530	609	0
03KBH1	1992	Jan-03	8,573	MW405342	370	35,800	2,526	609	0
03KBH1	1992	Jan-03	8,579	MW405343	370	35,800	2,538	609	0
04KBH8	1992	Aug-04	8,570	DQ054367	473	35,800	2,529	609	0

KSB; Korean subclade of subtype B, mo; month, y; year, Td; two drug combination therapy, cART; combinational antiretroviral therapy, ND; no data. HP: hemophiliacs. Near full-length and *env* gene; from *gag* gene to *nef* gene and *env* sequence length, respectively. [a]; FL is the presumed length based on only the sequence lengths of *env* and *nef* gene as other genes are normal size. [b]; The longest *nef* gene was 651-bp in 3 patients (89-17, HP-4 and HP-15) except a 799-bp with duplication of 77-bp and insertion of COA 1 homolog (71-bp) in patient PJA. [c]; contains a gross deletion.
