# Supplementary material for: Sequence Length of HIV-1 Subtype B Increases over Time: Analysis of a Cohort of Patients with Hemophilia over 30 Years
Source: Viruses. 2021 Apr 30;13(5):806. doi: 10.3390/v13050806 (PMC8145643; doi:10.3390/v13050806)
Supplement: Supplementary file 1 [file viruses-13-00806-s001.zip › Table S2.pdf]

Table S2. Signature pattern residues in the Pol proteins of 96 Korean subclade of HIV-1 subtype B.

**31 Korean signature nucleotides and 11 Korean signature amino acids residues**																
Position in NL43	**2362**	2366	2372	**2439**	2645	2684	2717	2840	3005	3023	3110	**3148**	**3282**	3287	3339	**3679**
	**A**	G	A	**C**	A	A	C	T	A	A	G	**C**	**G**	G	T	**C**
Western B (n = 31)	**A22**	G24	A19	**C20**	A30	G22	C29	C15	A28	A27	G30	**C30**	**G31**	G30	T31	**C23**
	**G4**	A3	G6	**A3**		A8	T1	T12	G2	G3	A1	**T1**		A1		**A8**
	**C1**			**T1**				A2								
								G1								
KSB (n = 96)	**G81**	A94	G90	**A74**	G93	A96	T93	A95	G90	G87	A94	**T76**	**A80**	A93	C90	**T73**
	**A15**	G2	A6	**G18**	A3		C3	T1	A6	A9	G2	**C16**	**G12**	G3	T3	**C19**
				**C4**								**G4**	**C4**			**A3**
																**G1**
KSAA*	**N37S**			**P63T**								**T299I**	**V345 M**			**T377I**

**31 Korean signature nucleotides and 11 Korean signature amino acids residues**															
Position in NL43	3776	3839	3896	**3951**	2959	4016	4106	**4268**	**4320**	4415	**4530**	**4600**	4859	4866	**4950**
	C	G	A	**C**	G	G	T	**G**	**G**	G	**C**	**C**	T	T	**C**
Western B (n = 31)	C31	G24	G23	**T18**	T22	A19	T30	**G17**	**G26**	A23	**A19**	**C31**	T30	T25	**C25**
		A7	A7	**C7**	G5	G11	C1	**A10**	**A4**	G8	**C10**		C1	C6	**T2**
			T1	**A6**	A4	T1		**T2**							
								**C1**							
KSB (n = 96)	T95	A92	A93	**C81**	G91	G82	C95	**A95**	**A89**	G94	**C76**	**A93**	C93	C96	**T74**
	C1	G4	G2	**T14**	A5	A14	T1	**G1**	**G7**	A2	**A19**	**C3**	T2		**C20**
			C1	**A1**							**T1**		A1		
KSAA[a]				**S468P**				**R672K**	**V690I**		**I760L**	**T783N**			**A900V**

In total, 31 residues were found to be Korean signature nucleotide residues based on the criteria (>75%) applied in a previous study [2]. All 11 Korean signatures amino acids were derived from minor amino acids from Western subtype B. This finding provides evidence for a founder effect because KSB was derived from its parent, Western subtype B [5,6]. Position in NL43* denotes from the initial codon of protease coding protein. In total, 11 Korean signature amino acids (KSAA)[a] originated from nonsynonymous changes from Western subtype B.
