# Supplementary material for: Sequence Length of HIV-1 Subtype B Increases over Time: Analysis of a Cohort of Patients with Hemophilia over 30 Years
Source: Viruses. 2021 Apr 30;13(5):806. doi: 10.3390/v13050806 (PMC8145643; doi:10.3390/v13050806)
Supplement: Supplementary file 1 [file viruses-13-00806-s001.zip › Table S3.pdf]

Table S3. Comparison of the frequency of the signature pattern nucleotides in 62 KSB-infected patients.

Gene	*gag*		*pol*		*vif*	*vpr,tat,rev,vpu*	*env*	*nef*
Nucleotide position	2190[a]	2321	4235	5070[a]	5253	None	6469	None
Signature	G	G		A				
Cluster O (n = 9)	1.0	1.0		1.0				
Other KSB (n = 53)	0.0	0.0		0.0				
Signature			G		A		G	
Cluster P (n = 13)			1.0		1.0		1.0	
Other KSB (n = 49)			0.0		0		0.0	

N = the number of patients infected with KSB, including 20 hemophiliacs and local controls. Positions 2190[a] and 5070[a] overlap with *pol* and *vif* gene, respectively. Nucleotide positions in the second row indicate the position of the nucleotides in the third and sixth rows in HIV-1 NL4-3. 1 and 0.0 denote 100% and 0% of all sequences within cluster O and other KSB, respectively [2-7].
