# Supplementary figures and images for: Sequence Length of HIV-1 Subtype B Increases over Time: Analysis of a Cohort of Patients with Hemophilia over 30 Years
Source: Viruses. 2021 Apr 30;13(5):806. doi: 10.3390/v13050806 (PMC8145643; doi:10.3390/v13050806)

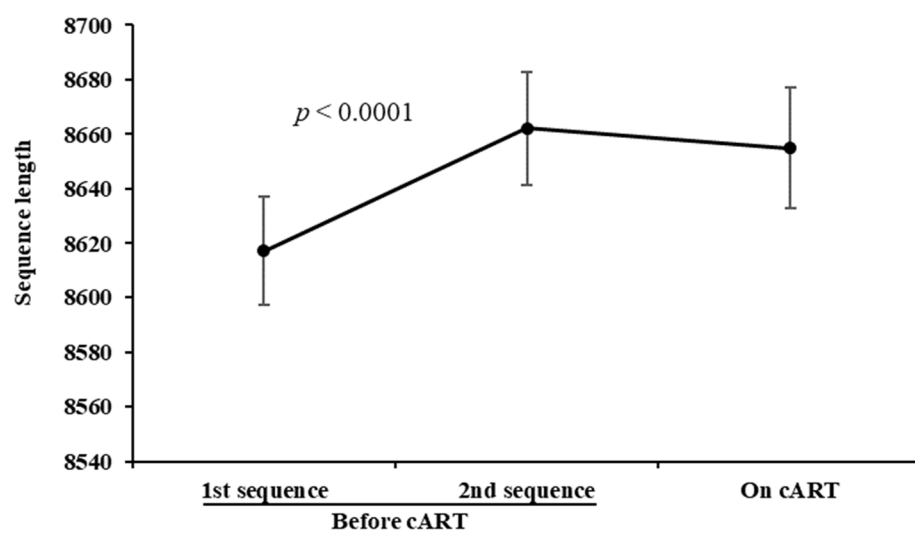

Figure S1

Supplement: Supplementary file 1 [file viruses-13-00806-s001.zip › Figure S1.pdf]
