# Supplementary material for: Sequence Length of HIV-1 Subtype B Increases over Time: Analysis of a Cohort of Patients with Hemophilia over 30 Years
Source: Viruses. 2021 Apr 30;13(5):806. doi: 10.3390/v13050806 (PMC8145643; doi:10.3390/v13050806)
Supplement: Supplementary file 1 [file Figure S2.pdf › Figure S2.pdf]

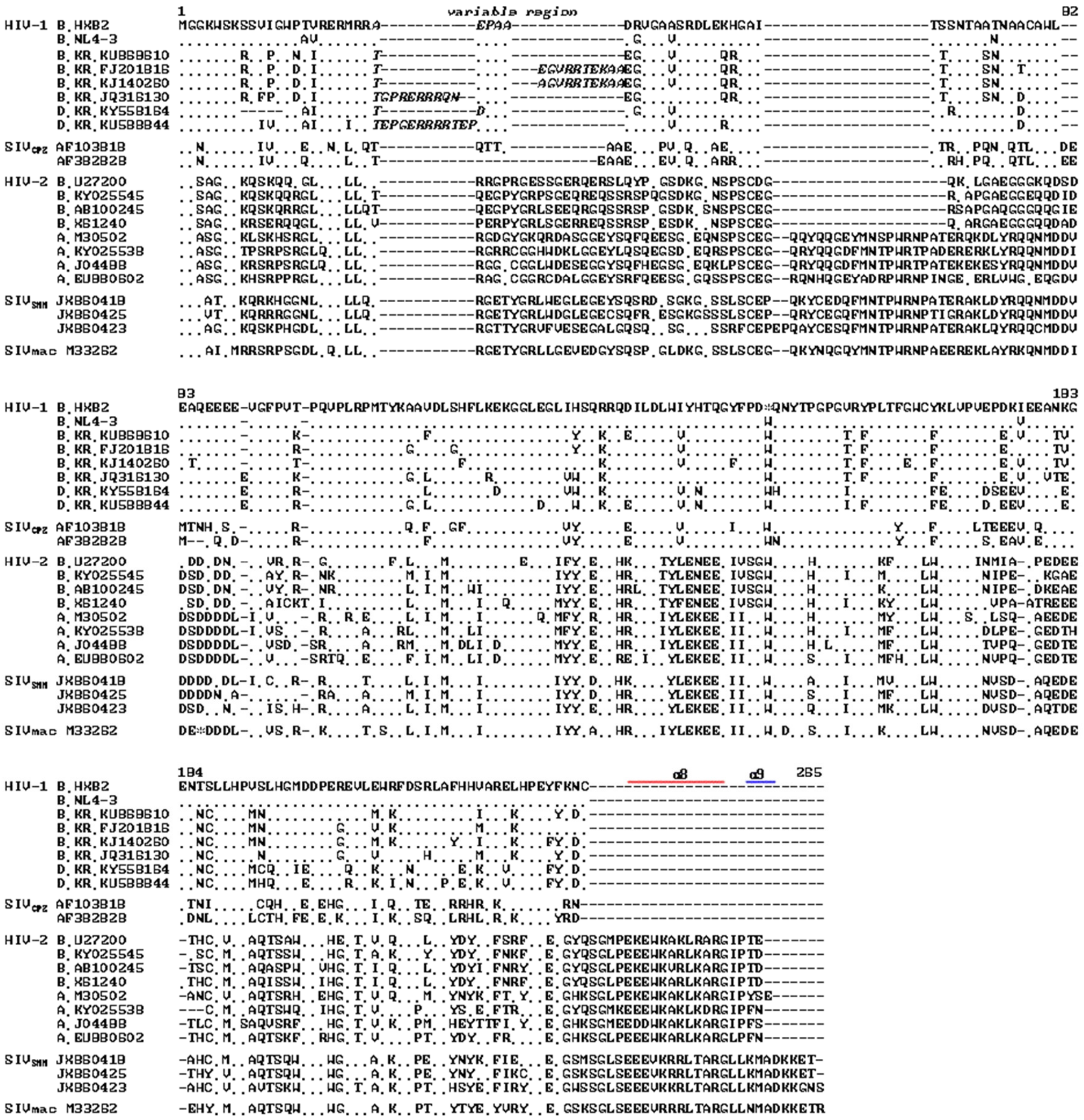
variable region
HIV-1 B.HXB2
B.NL4-3
B.KR.KU868610
B.KR.FJ201818
B.KR.KJ140260
B.KR.JQ316130
D.KR.KY558164
D.KR.KU588844
SIVcpz AF103818
AF382828
HIV-2 B.U27200
B.KY025545
B.AB100245
B.X61240
A.M30502
A.KY025538
A.J04498
A.EU880602
SIVsmm JX860418
JX860425
JX860423
SIVmac M33262
α8
α9

Figure S2
